# Paraldehyde

**Published:** 1893-08-19

**Authors:** 


### New Drugs and Preparations.

[We should be glad to receive, at our Office, 428, Strand, London, W.C., from the manufacturers, specimens of all new preparations and drugs which may be brought out from time to time.]

### PARALDEHYDE.

The use of paraldehyde as a hypnotic is very common in hospital practice, but in private, where the individual tastes of the patient necessarily receive more consideration, it appears to be but little employed, other more pleasant but probably less efficacious drugs being prescribed in its place. The fact that it is certainly one of the safest, and probably one of the most reliable of the ordinary hypnotics should, in the writer's opinion, outweigh its disadvantages and entitle it to a more extended use.

That it is a polymer of aldehyde, and therefore closely connected with the group of alcohols, explains the peculiar taste and smell which are the great drawbacks to its employment. Both are very penetrating, and of a somewhat alcoholic nature, but very decidedly nauseous and unpleasant. Partly for this reason, and partly because of the very limited extent to which paraldehyde is soluble in water, it is advisable when not for other reasons contra-indicated, to administer it in a little brandy or port wine, which will serve to somewhat drown its taste, and at the same time to ensure a more complete solution. If given in water alone, the drug on standing invariably separates out and sinks to the bottom of the glass; but a temporary and fairly complete mixture can be obtained by vigorously shaking for some seconds immediately before use. When prescribed with other drugs, it should be suspended in mucilage, but even with this precaution it is well to first shake the bottle in order to make sure that no separation has occurred. Finally, it can be obtained, if desired, in capsules; but the extremely small dose, namely ♏iii., contained in those usually sold, renders this a very unsuitable method of administration.

The chief use of paraldehyde is that of a simple hypnotic, and in cases of ordinary insomnia, especially when accompanying other diseases, it will be found to be a most reliable and valuable drug. It has the great advantage over sulphonal—which is probably one of the most widely used hypnotics in private practice—that its action is speedy, rarely requiring, in cases in which it is successful in inducing sleep, more than half an hour before it takes effect; whilst it is certainly the more reliable of the two drugs, and produces no unpleasant after-effects of any sort. When given for simple insomnia, the usual dose is ʒss., but in more severe cases this quantity may safely be doubled. When the insomnia is accompanied or caused by delirium or insanity, it is undoubtedly, in the writer's opinion, with the exception of chloral hydrate, the most efficacious hypnotic; whilst it is both safer and more pleasant in its effects than the latter drug. In such cases, however, large doses must be given, nothing less that ʒj. being likely to act, whilst even as much as ʒii. is sometimes necessary to produce sleep.

The use of paraldehyde appears to be practically devoid of danger, and testimony is afforded to its harmlessness in moderate doses by the fact that a case has been recorded in which recovery ensued after taking as much as 3½ oz. Exception must, however, possibly be made of cases of emphysema, Dr. Rolleston having recorded an instance of severe collapse and dyspnœa produced by it in this disease. Physiologically, it strengthens the heart and diminishes the frequency of its beat, whilst it considerably increases the flow of urine; and it is therefore peculiarly applicable to cases of heart disease accompanied by insomnia.

In addition to its use as a hypnotic, paraldehyde acts with considerable benefit in cases in which paroxysmal dyspnœa of pulmonary origin is a prominent symptom. Attention was some time ago called to this fact in the *British Medical Journal*; and the writer has since then frequently used this drug in cases of asthma or chronic bronchitis, with paroxysmal asthmatic attacks, or even in cases of the latter disease, in which constant dyspnœa with deficient secretion is a distressing feature. In such cases of chronic bronchitis the addition of ♏x. or ♏xv. of paraldehyde to the drug ordinarily used has rarely failed in his experience to give considerable relief; nor have any bad or unpleasant effects ever been produced. In asthma, however, like all other drugs used for this disease, the action is uncertain, and often either fails altogether or rapidly ceases; sometimes, on the other hand, very good results have ensued from its use. As already pointed out, the paraldehyde must be suspended in mucilage or some other substance, and the bottle well shaken before a dose is taken.

